# Pío del Río-Hortega: A Visionary in the Pathology of Central Nervous System Tumors

**DOI:** 10.3389/fnana.2016.00013

**Published:** 2016-03-01

**Authors:** Santiago Ramon y Cajal Agüeras

**Affiliations:** Pathology Department, Vall d'Hebron University Hospital, Universidad Autónoma of BarcelonaBarcelona, Spain

**Keywords:** histogenesis, gliomas, Rio Hortega, brain tumors, classification

## Abstract

The last 140 years have seen considerable advances in knowledge of central nervous system tumors. However, the main tumor types had already been described during the early years of the twentieth century. The studies of Dr. Pío del Río Hortega have been ones of the most exhaustive histology and cytology-based studies of nervous system tumors. Río Hortega's work was performed using silver staining methods, which require a high level of practical skill and were therefore difficult to standardize. His technical aptitude and interest in nervous system tumors played a key role in the establishment of his classification, which was based on cell lineage and embryonic development. Río Hortega's approach was controversial when he proposed it. Current classifications are not only based on cell type and embryonic lineage, as well as on clinical characteristics, anatomical site, and age.

## Introduction

I am satisfied to be able to summarize the contribution of Pío del Río Hortega to the field of neuropathology, in particular, tumors of the central nervous system. As a pathologist, I am keenly aware of the classifications of central nervous system tumors and can now provide a context for many of the contributions of Dr. Pío del Río Hortega.

As stated in other sections of this monograph, Río de Hortega was an illustrious character, both at the human level and at the scientific level. A self-taught man with an extraordinary knowledge of laboratory techniques, he was a leading scientific figure in the cities where he worked. The combination of expertise, dedication, and an undeniable persistence and capacity for work, together with considerable talent, led him to make key discoveries in the history of neuroscience, including microglia and oligodendroglia (as described in other chapters of this Special research Topic; see Pérez-Cerdá et al., 2015; Tremblay et al., 2015).

His techniques, especially the silver carbonate staining method, enabled him to work on tumors of the central nervous system and develop his highly practical classification (Polak, 1947; Llombart Rodríguez, 1965; Obrador, 1965).

## Historical context

The first descriptions of brain tumors date from the period of Virchow, who described gliomas arising from neuroglial cells. Virchow made a distinction between myxoglioma, gliosarcoma, glioma durum, and glioma hemorrhagicum, which are composed of glial cells that sometimes contained fibers. Virchow's pioneering comparison of neoplastic cells with normal brain tissue laid down the scientific foundations for all subsequent classifications of tumors of the central nervous system. The main studies at the end of the nineteenth century and beginning of the twentieth century were performed by Simon (1874), who described spider cell glioma, and Tooth and Conheim, who reported that tumors arose from embryonic remnants.

Between 1900 and 1950, the various classifications of central nervous system tumors led to decades of confusion over terminology. The more notable studies of the period were by Ribbert (1910, 1918) who speculated about the histogenesis and etiology of glioma, particularly in his paper on spongioblastoma and glioma (“Über das Spongioblastoma und das Gliom” [On spongioblastoma and glioma]). Some authors feel that this study had a negative effect on the classifications of glioma that were produced during the following 20 years. In his study, which was based on theoretical deductions, Ribbert concluded that differentiated glial areas can never return to a lower grade of differentiation and that gliomas, glioblastomas, and spongioblastomas would therefore necessarily have to be explained by the presence of embryonic remnants whose growth had stopped at various stages of differentiation. According to this hypothesis, Ribbert believed that tumors stemmed from embryonic stages and not from changes occurring in the most differentiated cells. Nevertheless, Ribbert paved the way for the cytological study of tumors and for more specific studies based on impregnation methods, of which Río Hortega was a major proponent. The histogenetic and embryological approach adopted by Ribbert was modified by the cellular approach espoused mainly by Río Hortega. The contributions of the French school (Lhermitte and Dumas, 1916; Cornil, 1924; Roussy and Oberling, 1932) around the 1920 made it possible to distinguish between fibrillary astrocytoma, four subgroups of non-fibrillary glioma (round, spindle-shaped, polymorphic, and ameboid cells), glioblastoma, and spongioblastoma. The classification included ependymomas with choroid plexus tumors, which were separate from the other gliomas. The histogenetic approach was maintained in the studies by Globus and Strauss (1925) and in those of Bailey and Cushing (1926), where a distinction is made between various histogenetic cell types in glioma. The authors recognize the considerable internal heterogeneity of these tumors, to the extent that their classification placed considerable emphasis on the predominant cell type. The same authors performed an exhaustive study of brain tumors based on morphologic characteristics and on correlations with the patient's prognosis after surgery (Bailey, 1924; Bailey and Bucy, 1929). Their classification developed from the concept that tumor cells could arise from a medullary epithelium parent cell, which could differentiate into other glial, neural, or choroid cells. These cells could then differentiate even further. In theory, tumors could develop at each of these phases of differentiation. This period saw the first description of oligodendromas and cerebellar medulloblastomas. Although these tumors had been described as sarcomas or neuroblastomas by other authors, Bailey and Cushing (1925) have the merit of separating them from neuroblastoma based on their gross appearance, origin, form of growth, and spread along the spinal cord.

The 1926 classification of Bailey and Cushing is similar to the present one. It distinguished between tumors of the central nervous parenchyma, as follows:

(1) Astrocytoma (grades I–IV), pilocytic astrocytoma, glioblastoma multiforme, oligodendroglioma, ependymoma, choroid plexus papilloma, pinealoma, colloid cyst, and medulloblastoma.
(2) Meningeal tumors: meningioma, malignant meningioma, meningeal sarcoma, and meningiomatosis.
(3) Tumors of the cranial pairs: neurinoma.
(4) Tumors of the pituitary gland: adenoma, invasive adenoma, carcinoma, and craniopharyngioma.
(5) Vascular tumors: hemangioblastoma.
(6) Embryonal tumors: dermoid cysts and teratomas.

This classification had an enormous impact on neuroscience and neurosurgery, although it was criticized by several authors, mainly Scherer, who stressed the lack of correlation between clinical and pathological aspects in several tumors and the fact that a very high number of tumors (up to 30%) could not be classified following the authors' criteria. In parallel, authors such as Cushing focused on clinical classifications that described tumors with a favorable prognosis, for example cerebellar astrocytoma, which they stressed was different from cerebral astrocytoma, despite the histogenetic similarity between the two. Using data from studies of the child brain, the authors described non-recurring cystic tumors that were well-defined in terms of growth stage and whose classification was highly relevant at the time. This distinction was not based merely on histogenetic and cytologic principles, but on clinical, histopathologic, and clinical data. Important as well the contributions of Penfield (1931).

The publications and lectures of Río Hortega during the 1930s played a major role in promoting histogenetic classification, largely thanks to very accurate silver staining techniques. Most authors from this period and thereafter felt that his classification contained the most exhaustive collection of images until then. Río Hortega's classification was not based on clinical findings or anatomical site, but on morphological and histogenetic data.

## Tumors of the central nervous system: the contribution of río hortega

During the initial stage of his training, Río Hortega analyzed brain tumors in four studies. One of these was his doctoral thesis (“Causas y Anatomía Patológica de los Tumores de Encéfalo” [Causes and Histopathology of Brain Tumors]), which he defended under the direction of his tutor, Leopoldo López García, between the years 1911 and 1912.

Río Hortega wrote papers on the histopathology of carcinomas and of the nervous system in patients with brain tumors (1911a), the pathophysiology of brain tumors (1911b), and abnormalities of nerve tissue and general symptoms of brain tumors (Río-Hortega, 1911a,b, 1912, 1914a,b,c; Río-Hortega and y Costero, 1928; Río-Hortega and y Álvarez Cascos, 1930).

During the following phase of his training, Río Hortega performed a study of subcutaneous giant cell glioma, the results of which were published in 1926 (Río-Hortega, 1926). The third phase (1928–1936) saw the appearance of his most important contributions to the field of neuro-oncology. In 1930, he published a series of monographs analyzing the cytologic and histogenetic characteristics of specific tumor groups, beginning with a detailed consideration of meningeal exotheliomas, which he discussed and included in the differential diagnosis of what was then known as Cushing meningioma. He described variations of meningioma, such as xanthomatous tumors and fascicular tumors, which have been reported sporadically by other authors. The examination of these histopathologic forms led him to propose three major patterns: (a) a predominantly syncytial pattern; (b) a pattern based on fibrillary differentiation of cytoplasm that tended to arrange itself in plaques and bundles; and (c) a pattern involving more epithelioid and lobulated morphological differentiation. Similarly, he described the formation of acervuli and psammoma bodies in meningioma, the pineal gland, and the colloid plexus (Río-Hortega, 1930c).

The year 1932 saw the publication of the major study “La estructura y sistematización de los gliomas y paragliomas” [Structure and systematization of gliomas and paragliomas], which, with more than 260 pages and 200 images, was the fruit of the techniques that Río Hortega had been developing using mainly silver carbonate staining (Río-Hortega, 1932, 1933a,b).

He performed the study using his in-depth knowledge of the histology of the glia and brain and had to seek the help of neurosurgeons and other pathologists to compile a sufficiently large series of brain tumor samples for study and classification. The French neurosurgeon Clovis Vincent was of inestimable help during this period.

The results, which were based on neuroembryological data, pointed to four potential evolutionary pathways of the primitive medullary epithelium (neuroblasts, glioblasts, pineoblasts, and choroideoblasts). Given the considerably heterogeneous nature of brain tumors, Río Hortega thought that it was important to classify them into histologic types with common embryological findings. Therefore, he tried to define two large groups of tumors, with emphasis on histological and embryological lineage. The first group comprised gliomas and the second paragliomas, which included all those tumors formed by immature or mature elements of the nervous system and tumors arising from choroidal folds and the pineal gland.

**Figure 1:**
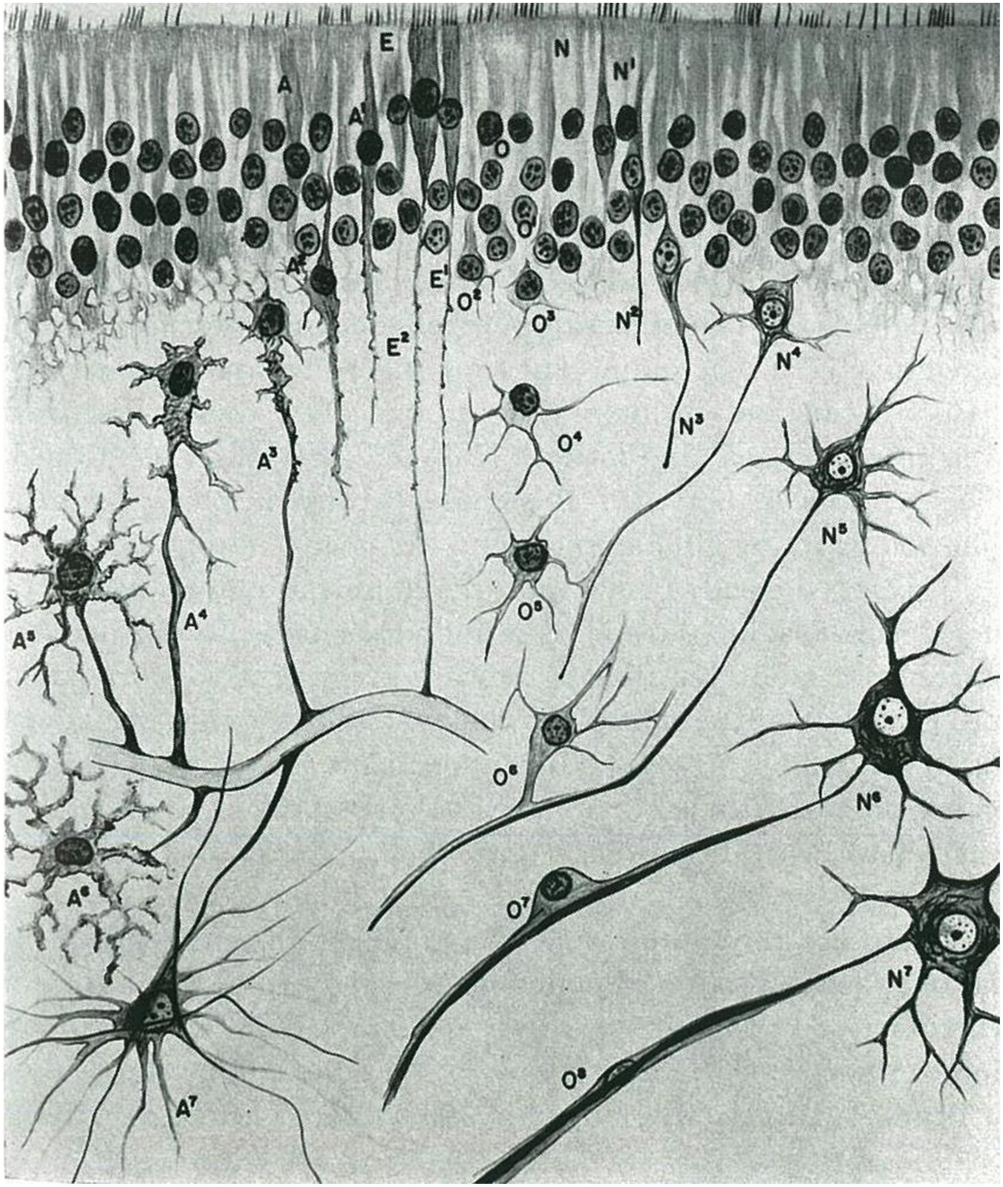
**Morphological evolution of the cells that are derived from the neural epithelium in the central nervous system (taken from Río-Hortega, 1933a)**.

**Figure 2:**
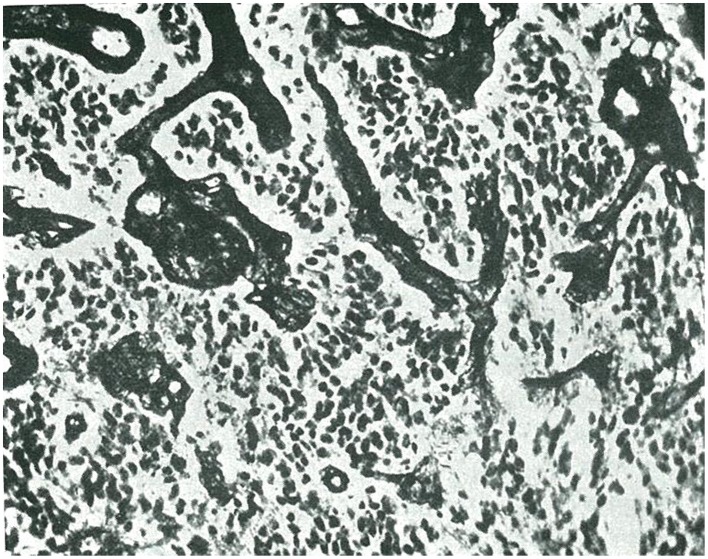
**Isomorphic glioblastoma, as termed by Rio-Hortega (taken from Río-Hortega, 1933a)**.

**Figure 3:**
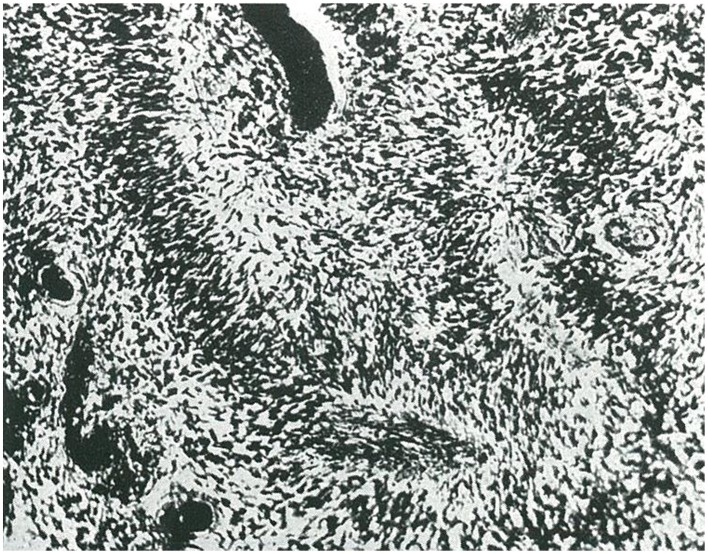
**Another example of isomorphic glioblastoma with wave-like arrangement of glioblasts (taken from Río-Hortega, 1933a)**.

**Figure 4:**
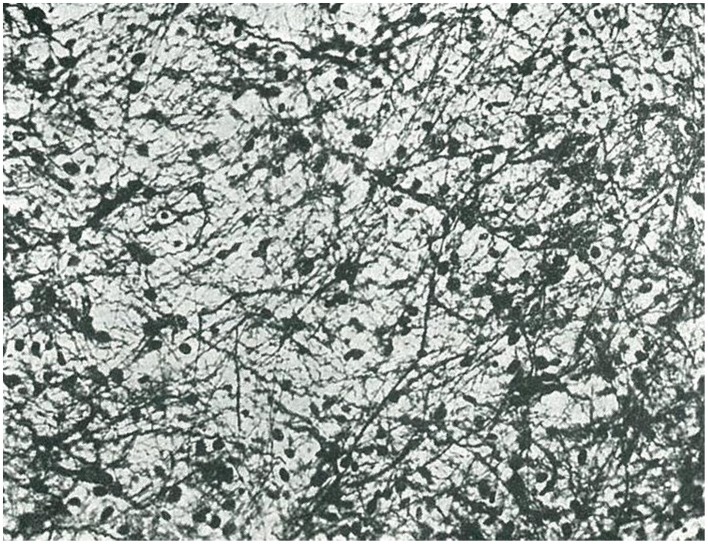
**Example of fibrous astrocytoma (taken from Río-Hortega, 1933a)**.

**Figure 5:**
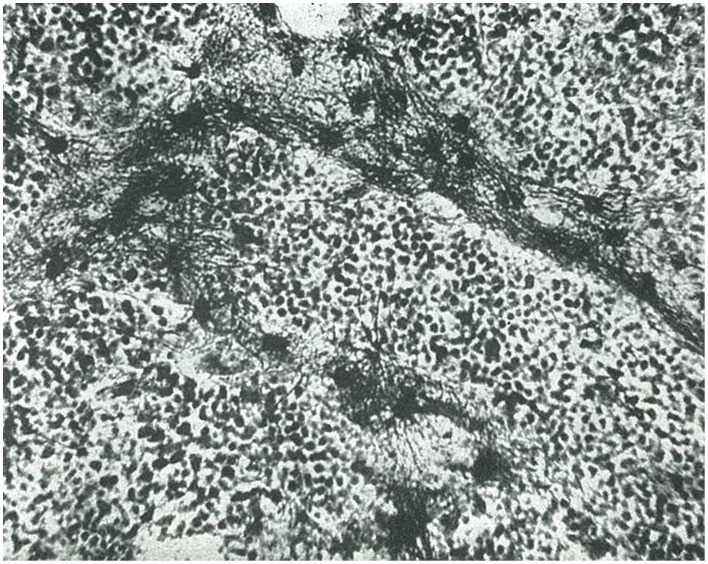
**Examples of oligodendrocytoma (taken from Río-Hortega, 1933a)**.

His systematic typing of the gliomas according to the degree of maturity of the cell components or the degree of differentiation enabled him to define the following entities (Río-Hortega and y Jiménez de Asúa, 1921; Río-Hortega, 1930a,b, 1932, 1933a,b; Pineda et al., 1962; Diaz, 1985) (Figure 1):

(1) Embryonal glioblastoma or spongioblastoma.
(2) Heteromorphic glioblastoma.
(3) Isomorphic glioblastoma (Figures 2, 3).
(4) Astroblastoma.
(5) Astrocytoma (Figure 4).
(6) Oligodendroglioma, with a distinction between oligodendrocytomas and oligodendroblastomas (Figure 5).
(7) Glioepitheliomas, which included ependymal tumors (ependymocytoma and ependymoblastoma).

**Figure 6:**
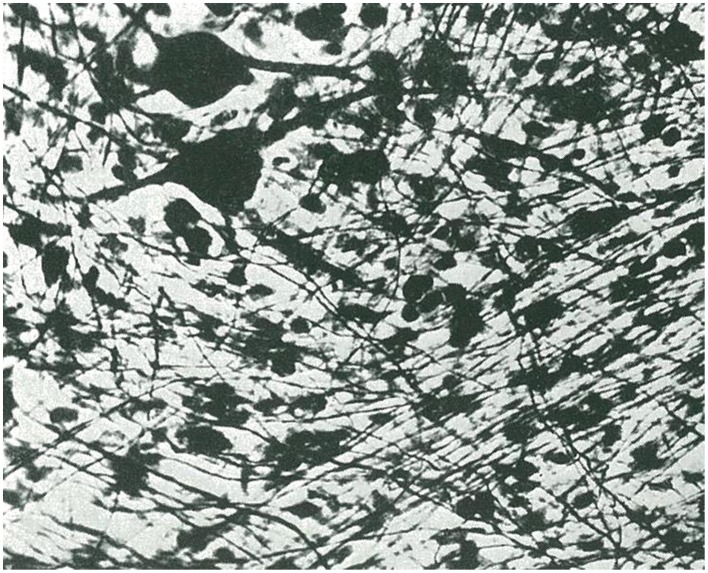
**Neurocytoma**. Note the gangliocytic cells and a plexus of unmyelinated fibers (taken from Río-Hortega, 1933a).

The classification of paragliomas included neuroma from neuroblasts (neuroblastoma), neurocytoma, pineal tumors (pinealcytoma, pineoblastoma), and choroid plexus tumors, which he termed chorioepitheliomas (Figure 6).

At the International Cancer Congress on the Scientific and Social Fight Against Cancer held in Madrid in 1933, he provided a more extensive summary of his classification in a lecture based on 287 pages of text (315 pages including the bibliography) and 248 images.

The classification, which to a certain extent complemented those proposed by Roussy and Overling and especially that proposed by Bailey and Cushing, differed in major areas, some of which are worthy of mention. Río Hortega's classification was based on the cytologic and embryologic characteristics of tumor cells, irrespective of their location, and therefore included tumors such as cerebellar medulloblastoma alongside tumors with a blastic lineage from within the brain. This distinction between medulloblastomas and other neuroblastic or primitive tumors was controversial at the time and continues to be so today.

**Figure 7:**
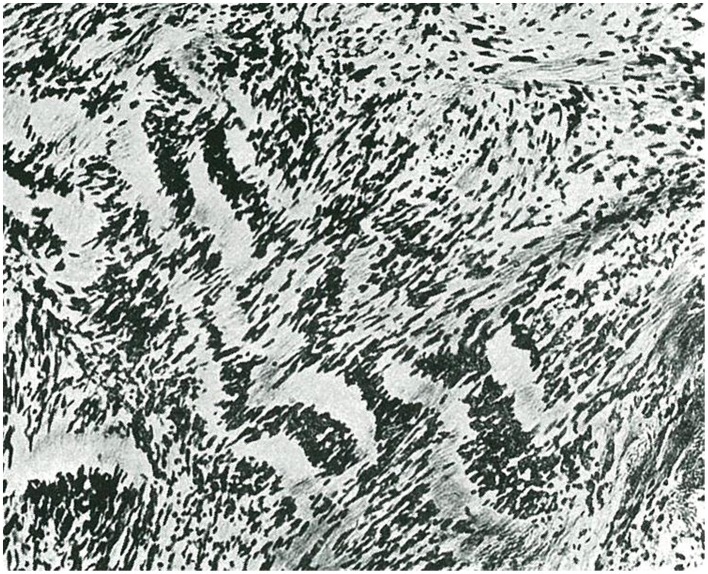
**Neurinoma**. Grouping of the nuclei in rows or palisades (taken from Río-Hortega, 1933a).

**Figure 8:**
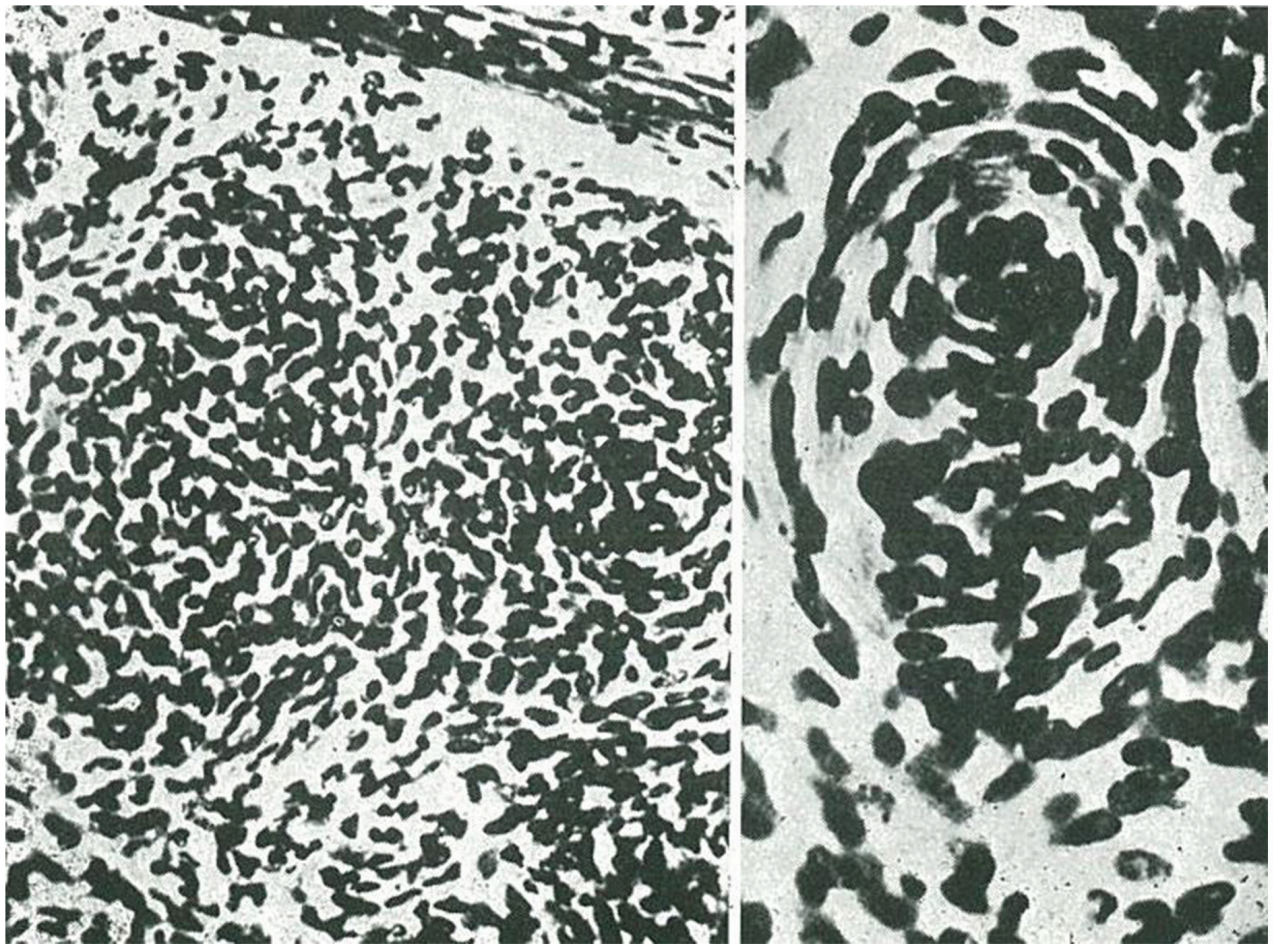
**Meningeal exothelioma**. The disordered cells are forming clusters and concentric layers or acervuli (taken from Río-Hortega, 1933a).

In his lecture, Río Hortega stressed the need for international harmonization of the nomenclature applied to tumors of the nervous system, as also proposed by Roussy and Overling. The groups he suggested in the lecture were as follows:

(1) Tumors arising from choroidal folds, the pineal valve, and homologous evaginations of the diencephalon that develop in the embryo.
(2) Tumors arising in the parenchyma of the brain and spinal cord and visual system, which is a prolongation of the brain.
(3) Tumors arising in the sympathetic nervous system, but not all those that develop from sympathogonia.
(4) Tumors arising in the nerve roots and in the peripheral nerves from interstitial cells or parenchymatous cells, depending on the interpretation of neoplastic elements (Figure 7).
(5) Tumors arising in the meninges owing to proliferation of cells or to new vascular formations (Figure 8).
(6) Tumors arising from hyperplasia in the parenchyma of the pituitary gland and from dislocated epidermal germ cells and invaginations of the Rathke pouch.

The fourth stage of Río Hortega's life (1936–1945), which was spent in exile, saw the publication of several papers on the nervous system (see below) (Río-Hortega, 1940a,b,c, 1941a,b, 1942, 1943).

In 1940, after his stay in Oxford, he performed a study on tumors of the optic nerve. During the same year, once he had settled in Argentina, he published a study on neuroblastomas, in which he concluded that there were no nervous system tumors with bipotential cells that were able to progress to neuroblasts or glioblasts and that most of the so-called medulloblastomas should be termed neuroblastomas, which is the term that corresponds to their lineage. His approach was considered scientific in terms of its embryological interpretation.

This interpretation remains controversial today. The embryological approach did not take histopathological characteristics into account. In addition, the neuroblastoma group included tumors that developed from other precursors, namely, medulloblasts, which develop in the molecular layer of the cerebellum.

His paper entitled “Del glioepitelioma al glioblastoma isomorfo” [From glioepithelioma to isomorphic glioblastoma], which was published in 1941, discussed and criticized the use of the term ependymoma—suggested by Bailey and Cushing—for tumors associated with the ependymal wall.

In 1943, Río Hortega (Río-Hortega et al., 1943) performed a cytological study of neurofibromas (also known as lemmocytomas), in which he described the histologic characteristics of the tumors and the elements that characterize them. He made an in-depth examination of the constitution of these tumors, discussed the identification of the main elements of Schwann cells and the embryonic origin thereof, and examined the specific differentiation of multiple neurofibromas and neurinomas (solitary schwannomas). His findings remain in force today.

He added new pathological information after previous papers (Cushing, 1917; Kernohan et al., 1931; Kernohan and Ody, 1932; Scherer, 1933).

In 1944, Río Hortega reported the results of an extensive study on oligodendrogliomas, which he classed as a gliomatous ectodermal variety characterized by small cells with a spherical nucleus (Río-Hortega, 1944a,b). His description of the nucleus as “very round” continues to be of use today in the diagnosis of oligodendrogliomas. Similarly, he observed that oligodendrocytes tend to be arranged in dense or diffuse patterns and never in perivascular patterns. Río Hortega established three cytological types of oligodendroglioma: (a) those whose cells have a spherical nucleus surrounded by a characteristic light halo and wrapped in a small layer of protoplasm that projects a varying number of fine and long appendages; (b) a more infrequent type of oligodendroglioma, which is formed by large neoplastic oligodendrocytes; and (c) a type that includes tumors with a non-uniform structure. As Río Hortega pointed out, the neoplastic oligodendrocyte evolves morphologically to the extent that it takes on the characteristics of an astrocytoma.

The year 1944 is also notable for Río Hortega's cytological study of tumors of the optic chiasm and nerve. The tumors described at this level that can be classed as gliomas, which were similar to brain tumors, with a moderately expansive or infiltrative character. The several cell types that can be identified for tumors of the optic nerve and chiasm include the following: (1) cells with small, round nuclei; (2) cells with bipolar, spindle-shaped, and long nuclei; (3) cells with a tripolar cytoplasm and thick prolongations; (4) cells with multipolar cytoplasm and fibroid and undulating prolongations; (5) cells with multipolar cytoplasm that invade the vasculature. Río Hortega reached the conclusion, albeit indefinite, that there are two basic neoplastic types in the formations he studied: one characterized by long elements (Schwann oligodendrocytes) and another defined by multipolar elements that give it the appearance of astrocytes (Ortiz de Picon, 1983).

## Classification of central nervous system tumors after río hortega

Current classifications of nervous system tumors are mixed, based on cytological and histogenetic criteria, as well as on histopathological variants that are of clinical and prognostic importance.

The main studies published after Río Hortega include that of Kernohan and Sayre (1952), Miller et al. (1952) who began to grade gliomas by establishing a correlation between microscopy findings, degree of malignancy, and prognosis.

In 1965, Zülch stressed the importance of other factors, such as patient survival, and included the concept of clinical malignancy (Zülch, 1965). Finally, the first classification of the World Health Organization was published in 1979 (Zülch, 1979) and classified tumors as follows:

(1) Tumors of neuroepithelial tissue, including astrocytoma, glioblastoma multiforme, oligodendroglioma, ependymoma, pinealcytoma, medulloblastoma, gangliocytoma, ganglioglioma, and neuroblastoma.
(2) Meningeal tumors, such as meningioma and meningeal sarcoma.
(3) Tumors of nerve sheath cells, such as neurinoma and neurofibroma.
(4) Primary cerebral lymphoma.
(5) Tumors arising in blood vessels, such as hemangioblastoma.
(6) Germ cell tumors, such as germinoma and teratoma.
(7) Metastatic tumors.
(8) Malformative tumors and tumor-like lesions, such as craniopharyngioma, epidermoid cyst, dermoid cyst, and colloid cyst of the third ventricle.
(9) Local extensions from regional tumors, such as glomus jugulare tumor and chordoma.
(10) Tumors of the anterior pituitary, such as pituitary adenoma.
(11) Unclassified tumors.

This classification serves as the basis for the subsequent editions of the World Health Organization classification until the year 2007 and the subgroups that are currently being incorporated. The most notable new additions are as follows:

(1) Variants of grade 1 astrocytomas, such as fibrillary, protoplasmic, and gemistocytic astrocytoma.
(2) Pilocytic astrocytoma as an independent entity.
(3) Subependymal giant cell astrocytoma.
(4) Astroblastoma.
(5) Anaplastic (malignant) astrocytoma.

A distinction is also made between oligodendroglial tumors and oligoastrocytic tumors [oligoastrocytomas and anaplastic (malignant) oligodendrogliomas].

Within the ependymal tumors and colloid plexus tumors, it is important to distinguish between variants of ependymomas, such as myxopapillary ependymoma, papillary ependymoma, subependymoma, and anaplastic ependymoma. At the level of the colloid plexus, we must distinguish between colloid plexus papilloma and colloid plexus carcinoma.

The neuronal tumors include variants such as gangliocytoma, ganglioglioma, ganglioneuroblastoma, gangliocytoma, anaplastic (malignant) ganglioglioma, and neuroblastoma.

Among the poorly differentiated and embryonal tumors it is important to identify glioblastoma (with its two subvariants, glioblastoma with a sarcomatous component and giant cell glioblastoma), medulloblastoma, medulloepithelioma, primitive polar spongioblastoma, and gliomatosis cerebri.

The classification covers tumors of the meningeal and related tissues, such as meningioma, with at least 11 morphological variants depending on the predominance of the Schwann, angiomatous, and papillary component. Similarly, the anaplastic (malignant) variant of meningioma is a distinct entity.

The classification still includes vascular tumors (e.g., hemangioblastoma and a malignant variant known as monstrocellular carcinoma), primary malignant lymphoma, and several variants of germ cell tumors. The previously cited group of malformative tumors and tumor-like lesions is extended to include enterogenic cysts, lipoma, hypothalamic neuronal hamartoma, nasal glial heterotopia (nasal glioma), as well as various vascular malformations (capillary telangiectasia, arteriovenous malformations, and Sturge-Weber disease).

The 2007 classification continues to include new entities, mainly anatomical-clinical conditions where it is very important to distinguish between gliomas with a high and low degrees of malignancy based on cytological criteria. The new types of low-grade glioma described include angiocentric gliomas, which are variants of glioneuronal tumors (e.g., rosette-forming or papillary tumors), and cytological variants of tumors of the anterior pituitary (e.g., pituicytoma and spindle cell oncocytoma). We can also distinguish between pilocytic tumors and their pilomyxoid variants, which have a poorer clinical prognosis (Louis et al., 2007).

In the coming years, it will be necessary to add the molecular abnormalities underlying the transformation and malignancy of these tumors. Our knowledge is expected to increase thanks to amplification of genes such as EGFR in glioblastoma, loss of alleles on chromosomes 1p and 19p in oligodendroglioma, and mutations in genes such as in p53 and IDH1 in low-grade astrocytoma that progresses to malignant astrocytoma. Intra- and inter-tumoral heterogeneity could be understood as resulting from cancer stem cells and the accumulation of various molecular abnormalities.

As has occurred with other types of tumor, especially lymphoma, whose classifications have for decades been based merely on morphological or clinical criteria, a joint approach to classification is probably the most suitable for clinical practice. Cytological abnormalities, location, and histopathological characteristics could facilitate a more in-depth study of the various types of tumor. It is important to remember that the major objective of any classification is that the information it provides be of use in clinical practice. Only thus can the patient receive the best and most personalized treatment possible.

## Author contributions

The author confirms being the sole contributor of this work and approved it for publication.

## Funding

Fondo de Investigaciones Sanitarias (11/00185), Redes Temáticas de Investigación Cooperativa en Salud (Ref. RD06/0020/1020).

### Conflict of interest statement

The author declares that the research was conducted in the absence of any commercial or financial relationships that could be construed as a potential conflict of interest.
