# Corrigendum: Pío del Río-Hortega: A Visionary in the Pathology of Central Nervous System Tumors

**DOI:** 10.3389/fnana.2017.00027

**Published:** 2017-04-03

**Authors:** Santiago Ramon y Cajal Agüeras

**Affiliations:** Pathology Department, Vall d'Hebron University Hospital, Universidad Autónoma of BarcelonaBarcelona, Spain

**Keywords:** histogenesis, gliomas, Rio Hortega, brain tumors, classification

In the title of the original article, the name “del Río-Hortega” was incorrectly misspelled as “del Río Ortega”.

The authors apologize for this error and state that this does not change the scientific conclusions of the article in any way.

The original article has been updated.

## Conflict of interest statement

The authors declare that the research was conducted in the absence of any commercial or financial relationships that could be construed as a potential conflict of interest.

